# Supplementary material for: Mega macromolecules as single molecule lubricants for hard and soft surfaces
Source: Nat Commun. 2020 May 1;11:2139. doi: 10.1038/s41467-020-15975-6 (PMC7195476; doi:10.1038/s41467-020-15975-6)
Supplement: Supplementary file 1 — Supplementary Information [file 41467_2020_15975_MOESM1_ESM.pdf]

Mega Macromolecules as Single Molecule Lubricants for Hard and Soft Surfaces

Parambath Anilkumar,[1,2#] Taylor B. Lawson,[3,4#] Srinivas Abbina,[1,2#] Janne T.A. Mäkelä,[4,5,6#] Robert C. Sabatelle,[6] Lily E. Takeuchi,[1,2] Brian D. Snyder,[4,6] Mark W. Grinstaff,[3,6,7,8]* and Jayachandran N. Kizhakkedathu[1,2,9,10]*

[1]Centre for Blood Research, Life Sciences Institute, The University of British Columbia, Vancouver, B.C., Canada
[2]Department of Pathology and Laboratory Medicine, The University of British Columbia, Vancouver, B.C., Canada.
[3]Department of Mechanical Engineering, Boston University, Boston, MA, USA.
[4]Center for Advanced Orthopaedic Studies, Beth Israel Deaconess Medical Center, Harvard Medical School, Boston, MA, USA.
[5]Biophysics of Bone and Cartilage, Department of Applied Physics, University of Eastern Finland, Kuopio, Finland
[6]Department of Biomedical Engineering, Boston University, Boston, MA, USA
[7]Department of Medicine, Boston University, Boston, MA, USA
[8]Department of Chemistry, Boston University, Boston, MA, USA.
[9]Department of Chemistry, The University of British Columbia, Vancouver, BC., Canada
[10]School of Biomedical Engineering, The University of British Columbia, Vancouver, BC., Canada

#These authors contributed equally
*These authors jointly supervised this work

* Corresponding authors

Jayachandran N. Kizhakkedathu
Email: jay@pathology.ubc.ca
Phone: 1-604-822-7085
Fax: 1-604-822-7742

Mark W. Grinstaff
Email: mgrin@bu.edu
Phone: 1-617-358-3429
Fax: 1-617-353-6466

Supplementary Information

Supplementary Discussion:

Stribeck curve plots COF vs Hersey number

A Stribeck curve plots COF vs Hersey number, the key variable in a Stribeck curve. The Hersey number, a dimensionless number, is derived from multiplying velocity (m/s) with viscosity (Pa.s = N.s/m2), and dividing by load per unit length (N/m). The lubrication in the presence of a fluid of incompressible materials has been well characterized with respect to specific modes, classically displayed on Stribeck curves, which defines the lubrication modes and is fundamental in understanding lubrication. In order to elucidate the relative contributions of the mega HPGs and other control lubricants to the different modes of lubrication, we determined the Stribeck curves for the metal on metal surface. Given the experimental constraints for the cartilage on cartilage surface experiments, we performed a more limited experiment.

Supplementary Table 1. Hydrodynamic diameters, degree of branching, and hydration of the mega HPGs and comparison with low molecular weight analogues.

Entry	M_w (Đ)	Degree of Branching[a]	Hydrodynamic diameter (nm)[b]	Hydration (number of water molecules per polymer)[c]
HPG-1	120 kDa (1.15)	0.56	9.20[3]	-
HPG-2	491 kDa (1.40)	0.57	12.60[4]	-
HPG-3	840 kDa (1.20)	0.56	20.4 ± 0.5	-
mega HPG-1	1300 kDa (1.20)	0.57	21.2 ± 0.4	68600
mega HPG-2	2900 kDa (1.20)	0.54	30.6 ± 0.6	125500
mega HPG-3	9300 kDa (1.40)	0.53	43.0 ± 0.4	389300

[a]Determined by quasi-elastic light scattering (QELS) analysis. [b]Measured by ^{13}C inverse gated NMR spectroscopy. [c]Determined by DSC.

Supplementary Table 2. Comparison of solubility of mega HPGs with other linear synthetic polymers

Entry	Sample (M_n)	Solubility (mg/mL)
1	mega HPG-2 (2.5 MDa)	380
2	PEO (8 MDa)	20
3	PEO (4 MDa)	25
4	PVA (0.205 MDa)	200

Supplementary Table 3. Statistics derived for Fig. 3a (Error bars represent standard deviation, N = 3 replicates; one-way ANOVA used to compare groups, statistical differences are defined as p<0.05 =*, p<0.01=**, p<0.0001 =****)

Tukey's multiple comparisons test	Mean Diff.	95.00% CI of diff.	Significant?	Summary	Adjusted P Value
7% mega-HPG-1 vs. 7% mega-HPG-2	-6.06E-05	-0.002929 to 0.002808	No	Ns	>0.9999
7% mega-HPG-1 vs. 7% mega-HPG-3	-4.66E-05	-0.002915 to 0.002822	No	Ns	>0.9999
7% mega-HPG-1 vs. BSF	-0.000474	-0.003343 to 0.002394	No	Ns	0.9994
7% mega-HPG-1 vs. 23% mega-HPG-1	-0.004145	-0.007014 to -0.001276	Yes	**	0.0024
7% mega-HPG-1 vs. 23% mega-HPG-2	-0.003445	-0.006314 to -0.0005762	Yes	*	0.0128
7% mega-HPG-1 vs. 23% mega-HPG-3	-0.02821	-0.03108 to -0.02534	Yes	****	<0.0001
7% mega-HPG-1 vs. Synvisc	-0.000948	-0.003816 to 0.001921	No	Ns	0.9509
7% mega-HPG-1 vs. Pennzoil	-0.000109	-0.004166 to 0.003948	No	Ns	>0.9999
7% mega-HPG-2 vs. 7% mega-HPG-3	0.000014	-0.002855 to 0.002883	No	Ns	>0.9999
7% mega-HPG-2 vs. BSF	-0.000414	-0.003282 to 0.002455	No	Ns	0.9998
7% mega-HPG-2 vs. 23% mega-HPG-1	-0.004084	-0.006953 to -0.001216	Yes	**	0.0028
7% mega-HPG-2 vs. 23% mega-HPG-2	-0.003384	-0.006253 to -0.0005156	Yes	*	0.0148
7% mega-HPG-2 vs. 23% mega-HPG-3	-0.02815	-0.03102 to -0.02528	Yes	****	<0.0001
7% mega-HPG-2 vs. Synvisc	-0.000887	-0.003756 to 0.001982	No	Ns	0.9659
7% mega-HPG-2 vs. Pennzoil	-4.83E-05	-0.004105 to 0.004009	No	Ns	>0.9999
7% mega-HPG-3 vs. BSF	-0.000428	-0.003296 to 0.002441	No	Ns	0.9997
7% mega-HPG-3 vs. 23% mega-HPG-1	-0.004098	-0.006967 to -0.001230	Yes	**	0.0027
7% mega-HPG-3 vs. 23% mega-HPG-2	-0.003398	-0.006267 to -0.0005296	Yes	*	0.0143
7% mega-HPG-3 vs. 23% mega-HPG-3	-0.02817	-0.03103 to -0.02530	Yes	****	<0.0001
7% mega-HPG-3 vs. Synvisc	-0.000901	-0.003770 to 0.001968	No	Ns	0.9628
7% mega-HPG-3 vs. Pennzoil	-6.23E-05	-0.004119 to 0.003995	No	Ns	>0.9999
BSF vs. 23% mega-HPG-1	-0.003671	-0.006539 to -0.0008020	Yes	**	0.0074
BSF vs. 23% mega-HPG-2	-0.002971	-0.005839 to -0.0001020	Yes	*	0.0395
BSF vs. 23% mega-HPG-3	-0.02774	-0.03061 to -0.02487	Yes	****	<0.0001
BSF vs. Synvisc	-0.000473	-0.003342 to 0.002395	No	Ns	0.9994
BSF vs. Pennzoil	0.000365	-0.003692 to 0.004422	No	Ns	>0.9999
23% mega-HPG-1 vs. 23% mega-HPG-2	0.0007	-0.002169 to 0.003569	No	Ns	0.9918
23% mega-HPG-1 vs. 23% mega-HPG-3	-0.02407	-0.02694 to -0.02120	Yes	****	<0.0001
23% mega-HPG-1 vs. Synvisc	0.003197	0.0003286 to 0.006066	Yes	*	0.0231

23% mega-HPG-1 vs. Pennzoil	0.004036	-2.097e-005 to 0.008093	No	Ns	0.0517
23% mega-HPG-2 vs. 23% mega-HPG-3	-0.02477	-0.02764 to -0.02190	Yes	****	<0.0001
23% mega-HPG-2 vs. Synvisc	0.002497	-0.0003714 to 0.005366	No	Ns	0.1149
23% mega-HPG-2 vs. Pennzoil	0.003336	-0.0007210 to 0.007393	No	Ns	0.1541
23% mega-HPG-3 vs. Synvisc	0.02726	0.02440 to 0.03013	Yes	****	<0.0001
23% mega-HPG-3 vs. Pennzoil	0.0281	0.02405 to 0.03216	Yes	****	<0.0001
Synvisc vs. Pennzoil	0.000839	-0.003218 to 0.004896	No	Ns	0.9973

Supplementary Table 4. Statistics derived for Fig. 4. (Error bars represent standard deviation, N = 3 or greater replicates; one-way ANOVA used to compare groups, statistical differences indicated by asterisk where p<0.05 =*, p<0.01=**, p<0.0001 =****)

Tukey's multiple comparisons test	Mean Diff.	95.00% CI of diff.	Significant ?	Summary	Adjusted P Value	
Synvisc vs. OA SF (mild)	0.01787	-0.04312 to 0.07887	No	ns	0.9954	A-B
Synvisc vs. OA SF (severe)	-0.07995	-0.1282 to -0.03172	Yes	****	<0.0001	A-C
Synvisc vs. BSF	0.0378	-0.003765 to 0.07936	No	ns	0.1058	A-D
Synvisc vs. PBS	0.02111	-0.01624 to 0.05847	No	ns	0.714	A-E
Synvisc vs. mega HPG-1 7%	0.01298	-0.03015 to 0.05611	No	ns	0.9944	A-F
Synvisc vs. mega HPG-1 23%	0.02934	-0.01379 to 0.07248	No	ns	0.4569	A-G
Synvisc vs. mega HPG-2 7%	0.01254	-0.03270 to 0.05778	No	ns	0.9971	A-H
Synvisc vs. mega HPG-2 23%	0.0133	-0.03194 to 0.05854	No	ns	0.9953	A-I
Synvisc vs. mega HPG-3 7%	0.03907	-0.004066 to 0.08220	No	ns	0.1089	A-J
Synvisc vs. mega HPG-3 23%	0.03842	-0.006813 to 0.08366	No	ns	0.1657	A-K
OA SF (mild) vs. OA SF (severe)	-0.09782	-0.1625 to -0.03312	Yes	***	0.0002	B-C
OA SF (mild) vs. BSF	0.01993	-0.03997 to 0.07982	No	ns	0.9879	B-D
OA SF (mild) vs. PBS	0.003239	-0.05382 to 0.06030	No	ns	>0.9999	B-E
OA SF (mild) vs. mega HPG-1 7%	-0.004894	-0.06589 to 0.05610	No	ns	>0.9999	B-F
OA SF (mild) vs. mega HPG-1 23%	0.01147	-0.04953 to 0.07247	No	ns	0.9999	B-G
OA SF (mild) vs. mega HPG-2 7%	-0.005332	-0.06784 to 0.05717	No	ns	>0.9999	B-H
OA SF (mild) vs. mega HPG-2 23%	-0.004572	-0.06708 to 0.05793	No	ns	>0.9999	B-I
OA SF (mild) vs. mega HPG-3 7%	0.02119	-0.03981 to 0.08219	No	ns	0.9833	B-J
OA SF (mild) vs. mega HPG-3 23%	0.02055	-0.04195 to 0.08306	No	ns	0.9889	B-K
OA SF (severe) vs. BSF	0.1177	0.07092 to 0.1646	Yes	****	<0.0001	C-D
OA SF (severe) vs. PBS	0.1011	0.05793 to 0.1442	Yes	****	<0.0001	C-E
OA SF (severe) vs. mega HPG-1 7%	0.09293	0.04470 to 0.1411	Yes	****	<0.0001	C-F
OA SF (severe) vs. mega HPG-1 23%	0.1093	0.06107 to 0.1575	Yes	****	<0.0001	C-G
OA SF (severe) vs. mega HPG-2 7%	0.09249	0.04237 to 0.1426	Yes	****	<0.0001	C-H
OA SF (severe) vs. mega HPG-2 23%	0.09325	0.04313 to 0.1434	Yes	****	<0.0001	C-I
OA SF (severe) vs. mega HPG-3 7%	0.119	0.07079 to 0.1672	Yes	****	<0.0001	C-J
OA SF (severe) vs. mega HPG-3 23%	0.1184	0.06825 to 0.1685	Yes	****	<0.0001	C-K

BSF vs. PBS	-0.01669	-0.05222 to 0.01884	No	ns	0.8838	D-E
BSF vs. mega HPG-1 7%	-0.02482	-0.06638 to 0.01674	No	ns	0.6443	D-F
BSF vs. mega HPG-1 23%	-0.008455	-0.05002 to 0.03311	No	ns	0.9998	D-G
BSF vs. mega HPG-2 7%	-0.02526	-0.06900 to 0.01849	No	ns	0.6879	D-H
BSF vs. mega HPG-2 23%	-0.0245	-0.06824 to 0.01925	No	ns	0.7249	D-I
BSF vs. mega HPG-3 7%	0.001267	-0.04030 to 0.04283	No	ns	>0.9999	D-J
BSF vs. mega HPG-3 23%	0.000626	-0.04312 to 0.04437	No	ns	>0.9999	D-K
PBS vs. mega HPG-1 7%	-0.008133	-0.04549 to 0.02922	No	ns	0.9996	E-F
PBS vs. mega HPG-1 23%	0.008231	-0.02912 to 0.04559	No	ns	0.9996	E-G
PBS vs. mega HPG-2 7%	-0.008571	-0.04834 to 0.03120	No	ns	0.9997	E-H
PBS vs. mega HPG-2 23%	-0.00781	-0.04758 to 0.03196	No	ns	0.9999	E-I
PBS vs. mega HPG-3 7%	0.01795	-0.01940 to 0.05531	No	ns	0.8682	E-J
PBS vs. mega HPG-3 23%	0.01731	-0.02245 to 0.05708	No	ns	0.925	E-K
mega HPG-1 7% vs. mega HPG-1 23%	0.01636	-0.02677 to 0.05950	No	ns	0.969	F-G
mega HPG-1 7% vs. mega HPG-2 7%	-0.000438	-0.04568 to 0.04480	No	ns	>0.9999	F-H
mega HPG-1 7% vs. mega HPG-2 23%	0.000322	-0.04492 to 0.04556	No	ns	>0.9999	F-I
mega HPG-1 7% vs. mega HPG-3 7%	0.02609	-0.01705 to 0.06922	No	ns	0.6271	F-J
mega HPG-1 7% vs. mega HPG-3 23%	0.02544	-0.01979 to 0.07068	No	ns	0.7197	F-K
mega HPG-1 23% vs. mega HPG-2 7%	-0.0168	-0.06204 to 0.02844	No	ns	0.9732	G-H
mega HPG-1 23% vs. mega HPG-2 23%	-0.01604	-0.06128 to 0.02920	No	ns	0.9807	G-I
mega HPG-1 23% vs. mega HPG-3 7%	0.009722	-0.03341 to 0.05285	No	ns	0.9995	G-J
mega HPG-1 23% vs. mega HPG-3 23%	0.009081	-0.03616 to 0.05432	No	ns	0.9998	G-K
mega HPG-2 7% vs. mega HPG-2 23%	0.000761	-0.04649 to 0.04801	No	ns	>0.9999	H-I
mega HPG-2 7% vs. mega HPG-3 7%	0.02652	-0.01871 to 0.07176	No	ns	0.6683	H-J
mega HPG-2 7% vs. mega HPG-3 23%	0.02588	-0.02137 to 0.07313	No	ns	0.75	H-K
mega HPG-2 23% vs. mega HPG-3 7%	0.02576	-0.01947 to 0.07100	No	ns	0.7048	I-J
mega HPG-2 23% vs. mega HPG-3 23%	0.02512	-0.02213 to 0.07237	No	ns	0.7816	I-K
mega HPG-3 7% vs. mega HPG-3 23%	-0.000642	-0.04588 to 0.04460	No	ns	>0.9999	J-K

Supplementary Table 5. COF for other lubricants

Lubricant	System specifics	COF	References
Lubricin	Bovine cartilage on glass; 40% compressive strain imposed on each plug to ensure boundary mode, and 60min allowed for hydrostatic pressure to equilibrate	0.093	https://doi.org/10.1002/jor.23370
Inflammed SF 24 ^{o}C	Human cartilage on cartilage; 30N load, 1 mm/s speed	0.109 (static)/ 0.089 (dynamic)	10.1007/s11249-006-9069-11
Inflammed SF 37 ^{o}C	Human cartilage on cartilage; 30N load, 1 mm/s speed	0.06 (static)/ 0.055 (dynamic)	10.1007/s11249-006-9069-12
Water	Human cartilage on cartilage; 30N load, 1 mm/s speed	0.059 (static)/ 0.05 (dynamic)	10.1007/s11249-006-9069-13
NaCl 15mM	Human cartilage on cartilage; 30N load, 1 mm/s speed	0.07 (static)/ 0.06 (dynamic)	10.1007/s11249-006-9069-14
NaCl 150mM	Human cartilage on cartilage; 30N load, 1 mm/s speed	0.089 (static)/ 0.067 (dynamic)	10.1007/s11249-006-9069-15
PBS	Early OA human cartilag on cartilage, 6.27 N load, 1 mm/s sliding speed, 900s test duration	0.134 +/- 0.034	doi: 10.1016/j.joca.2009.03.020
PBS	Late OA human cartilag on cartilage, 6.27 N load, 1 mm/s sliding speed, 900s test duration	0.106 +/- 0.053	doi: 10.1016/j.joca.2009.03.021
Human SF	Early OA human cartilag on cartilage, 6.27 N load, 1 mm/s sliding speed, 900s test duration	0.04 +/- 0.018	doi: 10.1016/j.joca.2009.03.022
Human SF	Late OA human cartilag on cartilage, 6.27 N load, 1 mm/s sliding speed, 900s test duration	0.042 +/- 0.015	doi: 10.1016/j.joca.2009.03.023
PBS	Early OA human cartilag on glass, 6.27 N load, 1 mm/s sliding speed, 3600s test duration	0.026 +/- 0.009	doi: 10.1016/j.joca.2009.03.024
PBS	Late OA human cartilag on glass, 6.27 N load, 1 mm/s sliding speed, 3600s test duration	0.024 +/- 0.009	doi: 10.1016/j.joca.2009.03.025
Human SF	Early OA human cartilag on glass, 6.27 N load, 1 mm/s sliding speed, 3600s test duration	0.02 +/- 0.07	doi: 10.1016/j.joca.2009.03.026

Human SF	Late OA human cartilag on glass, 6.27 N load, 1 mm/s sliding speed, 3600s test duration	0.19 +/- 0.001	doi: 10.1016/j.joca.2009.03.027
Poly-oxanorbornane carboxylate	Bovine cartilage on cartilage; torsional friction for 10,080 rotations at 22 mm/s, 0.78 MPa compressive strain	0.015 ± 0.01	https://doi.org/10.1016/j.biomaterials.2018.08.009
Poly-oxanorbornane carboxylate containing pendent triethylene glycol chains	Bovine cartilage on cartilage; torsional friction for 10,080 rotations at 22 mm/s, 0.78 MPa compressive strain	0.0316 ± 0.0005	https://doi.org/10.1021/acsbiomaterials.9b00085
High-molecular-weight lubricious polymer with poly(2-methyl-2-oxazoline) side chains	Immature bovine cartilage mounted on a ball-on-disc microtribometer; 5 mm/s speed; 0.5, 0.7 and 0.9 MPa pressure for 10 minutes each	0.02 - 0.1	DOI: 10.1002/anie.201712534
Lubricin mimetic - chondroitin sulfate backbone with type II collagen and HA binding peptides	Trypsin treated cartilage on glass with lubricin mimetic AND purified, commercially availabl HA; 0.0873 rad/sec sliding speed; 2 min test duration	0.1 - 0.3	DOI: 10.1016/j.biomaterials.2015.09.012
pAA:PEG lubricant library with varying backbone lengths and ratios of the two polymers	Bovine cartilage on XX; 40% compressive strain impossed on each plug to ensure boundary mode, and 60min allowed for hydrostatic pressure to equilibrate	0.14 - 0.248	https://doi.org/10.1002/jor.23370
sodium poly(7-oxanorbornene-2-carboxylate)	Cartilage on cartilage equilibrated for 60 min and then subjected to a relative rotation of +2 revolutions (720°) at 5 deg/s (an effective velocity of 0.3 mm/ s)	0.06+-0.02	https://pubs.acs.org/doi/abs/10.1021/ja400695h

Supplementary Figures:

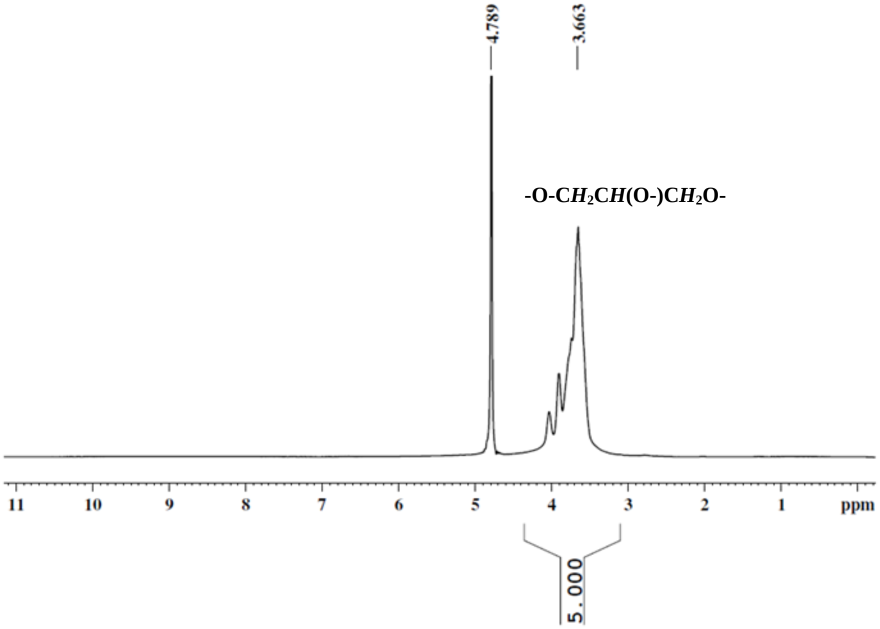

Supplementary Figure 1. ^{1}H NMR spectrum (D_2O, 400 MHz) of the HPG macroinitiator.

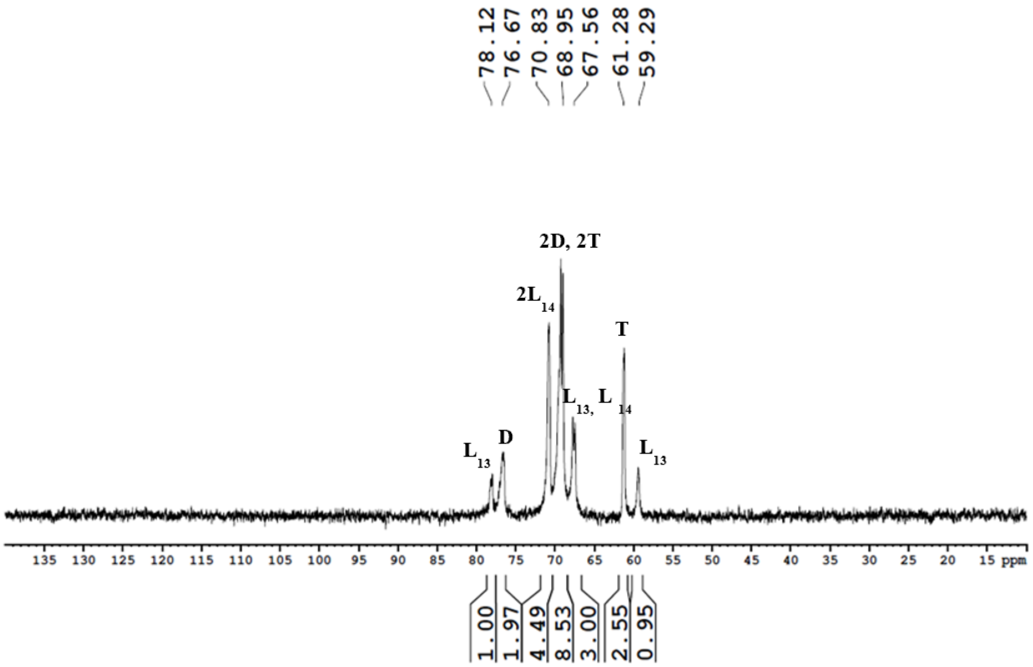

Supplementary Figure 2. ^{13}C NMR spectrum (D_2O, 100 MHz) of the HPG macroinitiator.

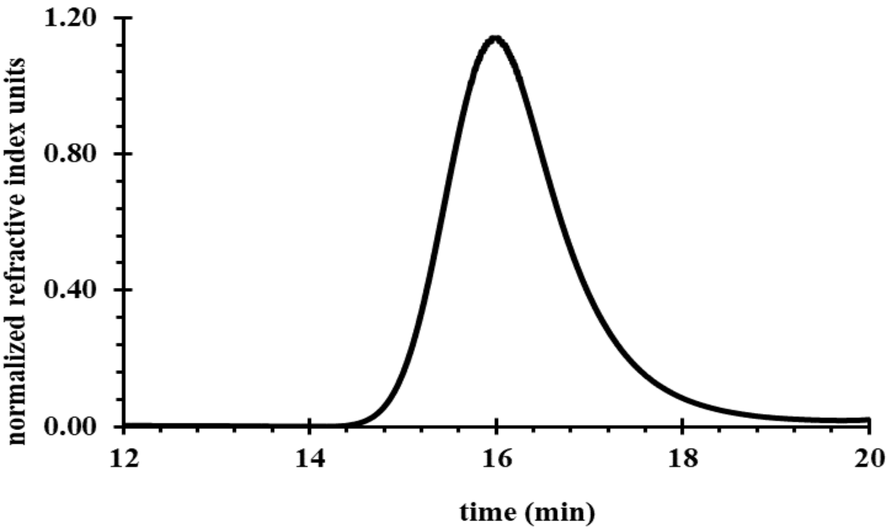

Supplementary Figure 3. GPC-MALS chromatogram (0.1 M $NaNO_3$ buffer, pH 7.4) of the HPG macroinitiator.

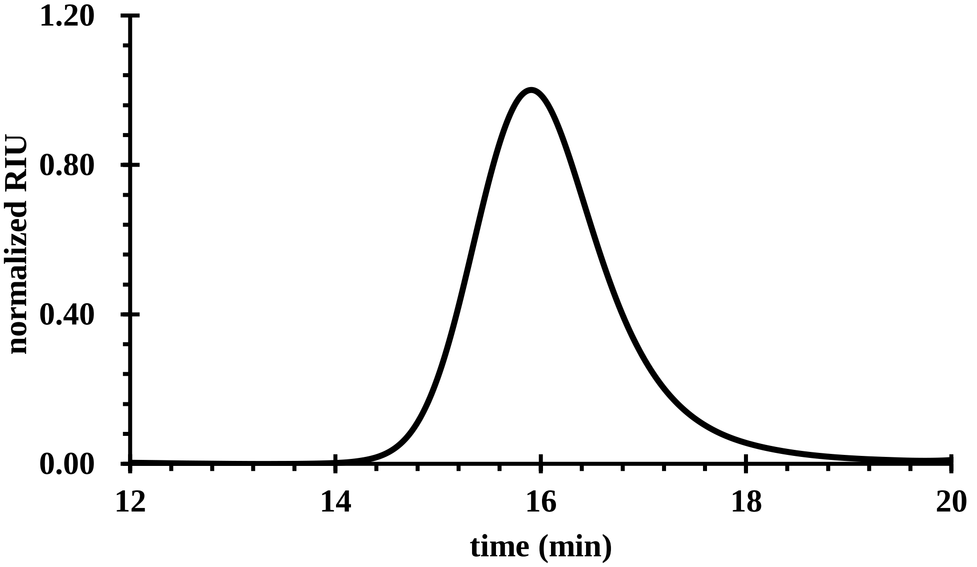

Supplementary Figure 4. GPC-MALS chromatogram (0.1 M $NaNO_3$ buffer, pH 7.4) of the mega HPG-1 (M_w-1.3 MDa).

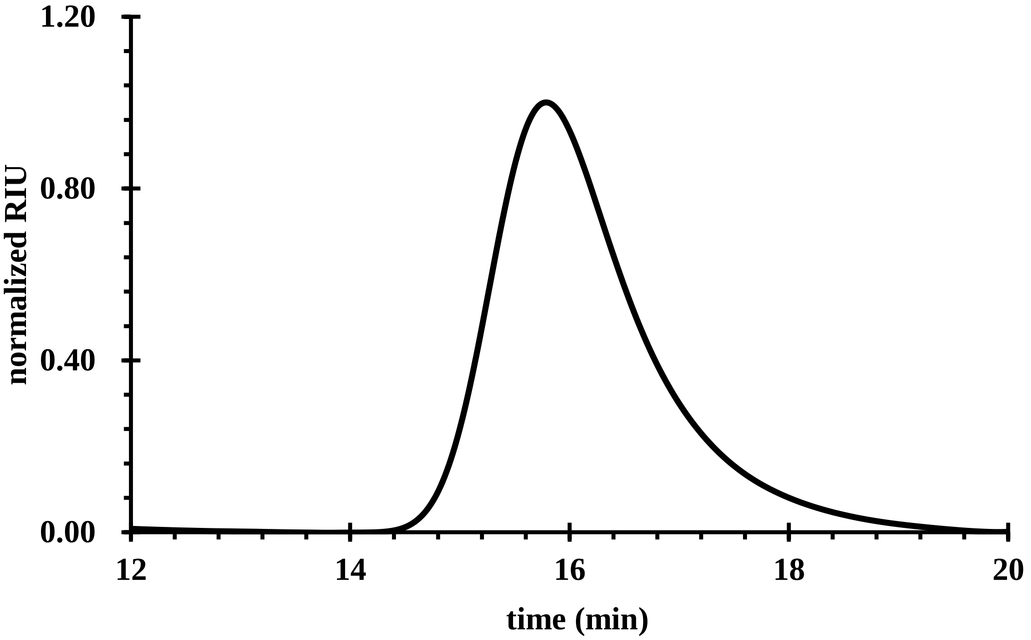

Supplementary Figure 5. GPC-MALS chromatogram (0.1 M $NaNO_3$ buffer, pH 7.4) of the mega HPG-2 (M_w-2.9 MDa).

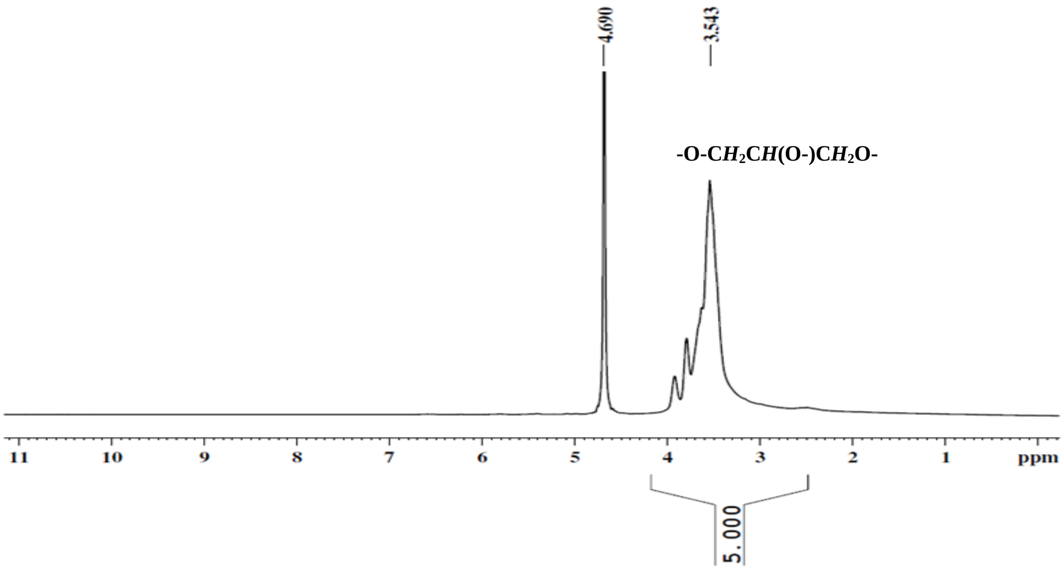

Supplementary Figure 6. ^{1}H NMR spectrum (D_2O, 400 MHz) of the mega HPG-1 (M_w-1.3 MDa).

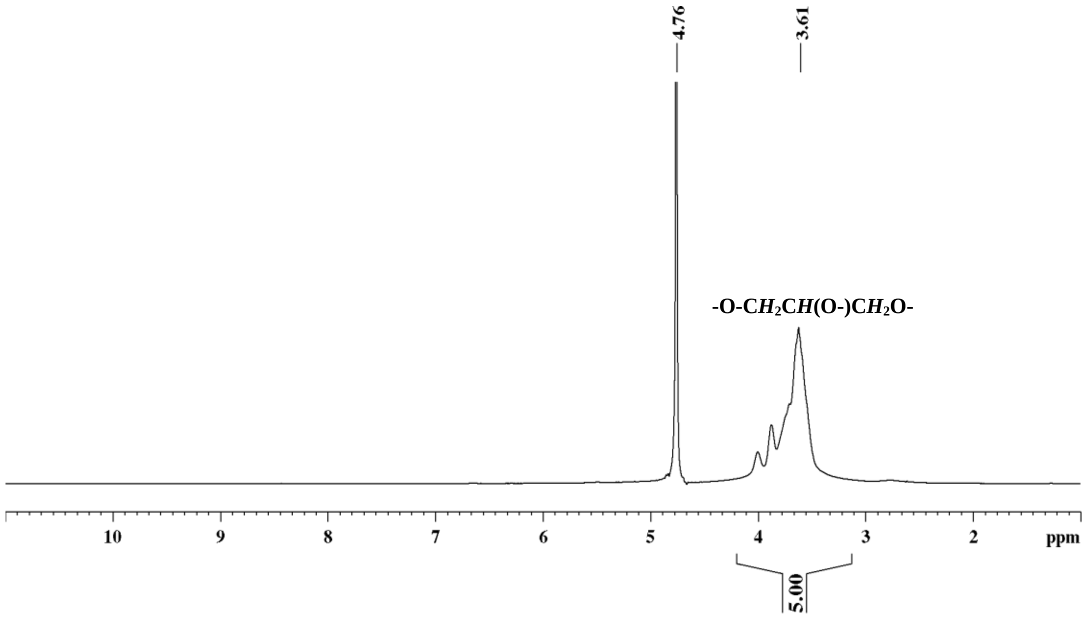

Supplementary Figure 7. ^{1}H NMR spectrum (D_2O, 400 MHz) of the mega HPG-2 (M_w-2.9 MDa).

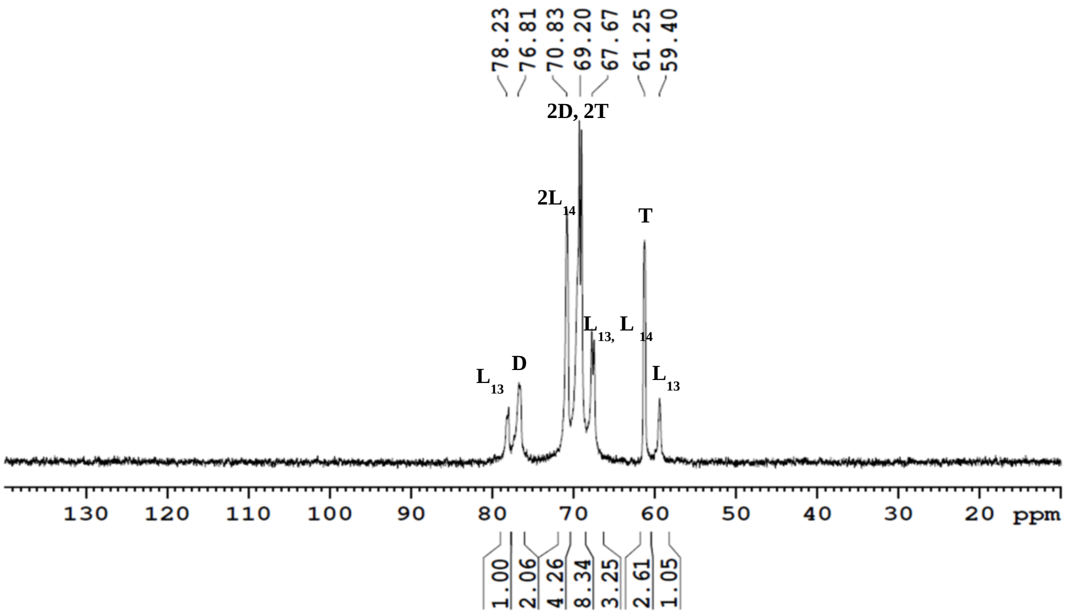

Supplementary Figure 8. ^{13}C NMR spectrum (D_2O, 100 MHz) of the mega HPG-1 (M_w-1.3 MDa).

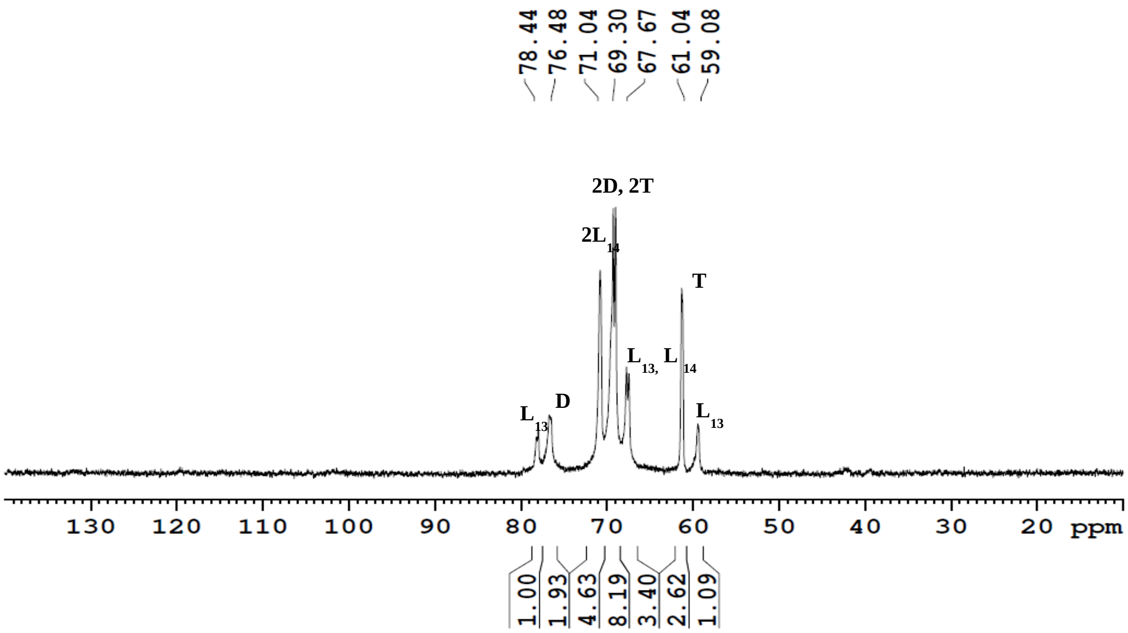

Supplementary Figure 9. ^{13}C NMR spectrum (D_2O, 100 MHz) of the mega HPG-2 (M_w-2.9 MDa).

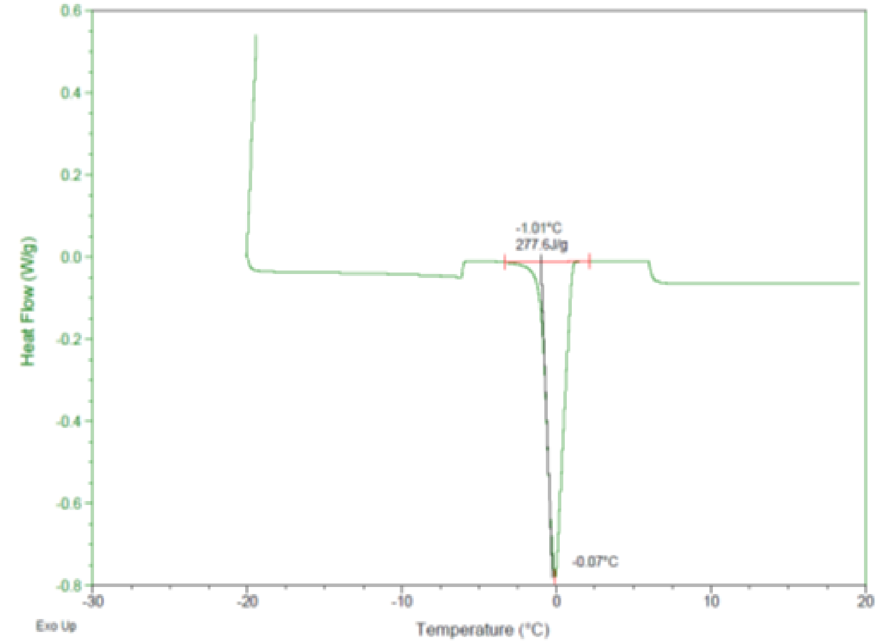

Supplementary Figure 10. Differential scanning calorimetry (DSC) thermogram of the mega HPG-3.

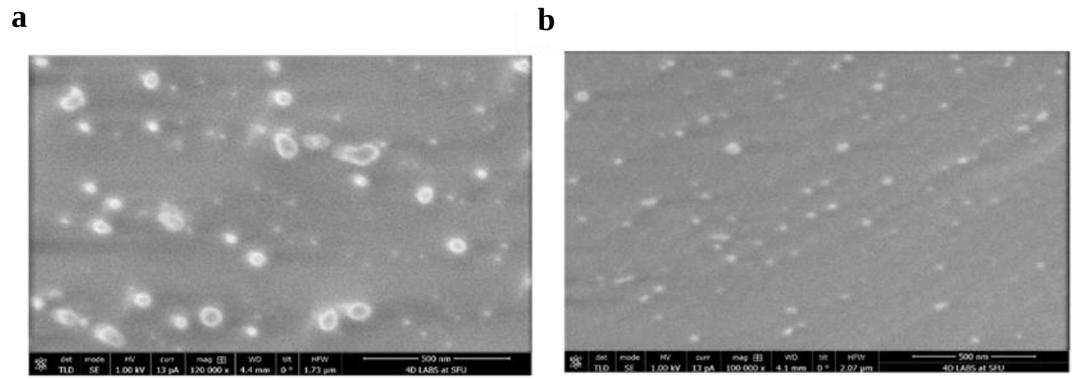

Supplementary Figure 11. Cryo SEM images of mega HPG-1 (**a**) and mega HPG-2 (**b**)

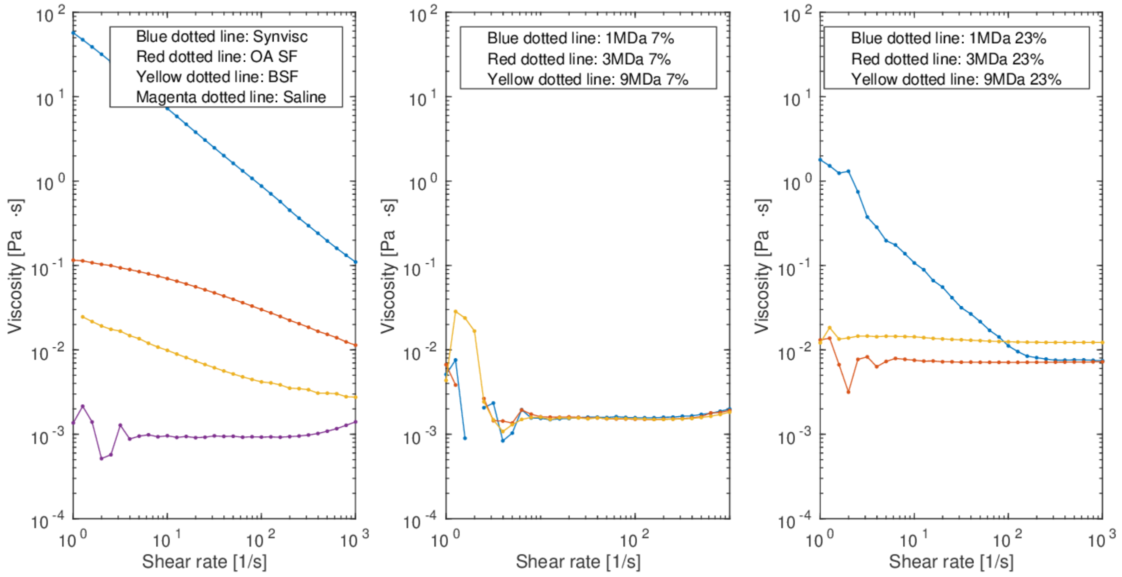

Supplementary Figure 12. The viscosity-shear rate behavior of mega HPGs at two different concentrations (7 and 23 wt%) and compared with Synvisc One (Synvisc), osteoarthritic synovial fluid (OA SF), bovine synovial fluid (BSF), and saline.

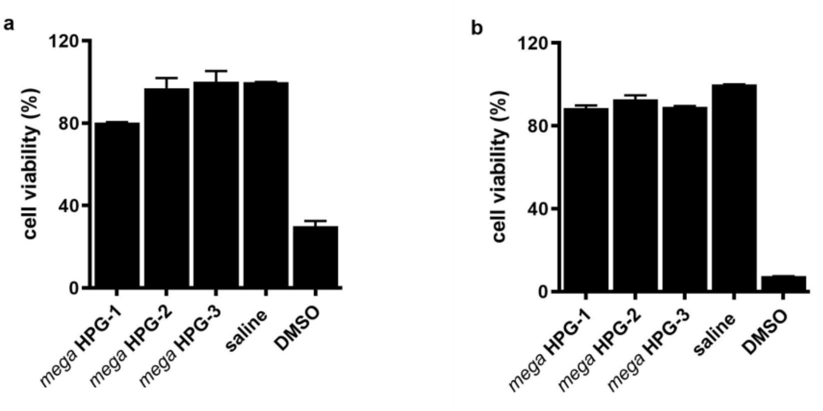

Supplementary Figure 13. Cell viability of mega HPGs-1, 2, and 3 (1.25 mg/ml) towards Tc28a2 juvenile human chondrocytes (**a**) and 3T3 murine fibroblast cells (**b**). Cells were incubated with either mega HPGs, saline, or DMSO for 48 h at 37 °C. After washings, the metabolic activity of the cells was assessed by MTT assay. Six replicates were performed and each study was repeated in quadruplicates. Average values and standard deviation are reported. Cell viability of mega HPGs (≥80%) irrespective of the cell line confirmed the high cell compatibility of the mega HPGs.

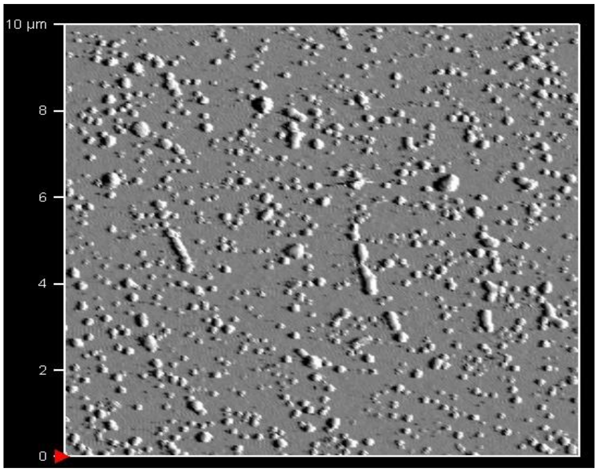

Supplementary Figure 14. AFM image of mega HPG-3 chemically adhered to the surface of an epoxide functionalized glass slide in 2D.
